# The application of pulmonary valve biorifice for reconstruction of right ventricular outflow tract in tetralogy of Fallot

**DOI:** 10.1186/1749-8090-8-152

**Published:** 2013-06-11

**Authors:** Jinfu Yang, Wenwu Zhou, Li Xie, Lian Xiong, Xin Wang, Yifeng Yang

**Affiliations:** 1 Department of the Cardiothoracic Surgery of the 2nd Xiangya Hosptial, Central South University, Middle Renmin road 139, 410011, Changsha, China; 2 Department of the Cardiothoracic Surgery of the Hunan Provincial People’s Hospital, Middle Jiefang Road 61, 410005, Changsha, China

**Keywords:** Tetralogy of Fallot, Pulmonary Valve, Prosthesis, Biorifice, Outcomes

## Abstract

**Background:**

To introduce a new technique to create a pulmonary valve biorifice for the reconstruction of the right ventricular outflow tract in tetralogy of Fallot (TOF), and to summarize the initial clinical experiment.

**Methods:**

The new technique of reconstructing the right ventricular outflow tract with pulmonary valve biorifice was used in 53 cases of TOF (the observation group). While the conventional technique for reconstructing the right ventricular outflow tract was used in other 50 cases of TOF (the control group). The clinical Data of all cases was reviewed retrospectively.

**Results:**

The ages, weights, cardiopulmonary bypass time, cardiac arrest time, as well as the post operation ventilation support time were not different significantly between groups. Unlike patients in the control group, patients from the observation group had shorter duration of ICU stay. Post- operation, in the observation group, only 2 cases had a large amount of pleural effusion, 1 case had mid-level effusion and 8 cases had a small amount of pleural effusion. While in the control group, there was 1 case of a large amount of effusion, 5 cases of mid-level effusion and 17 cases of a small amount of pleural effusion. 1 week after the operation, all patients were rechecked by echocardiography and no evidence of pulmonary valve stenosis was found. In the observation group, moderate pulmonary valve regurgitation was found in 8 cases, and mild regurgitation was observed in 15 cases. In the control group, severe regurgitation was observed in 3 cases, moderate regurgitation in 17 cases, and mild regurgitation in 16 cases. 33 cases from the observation group were rechecked six months, post-operation, and moderate-mild pulmonary regurgitation was found in 3 cases. As a follow up, 18 cases from the observation group were rechecked 1 year later, and no pulmonary regurgitation was found.

**Conclusion:**

The new technique to create pulmonary valve biorifice can reduce the pulmonary valve regurgitation, reduce postoperative pleural effusion, and improve the early surgical outcome.

## Background

Tetralogy of Fallot (TOF) is one the most common cyanotic congenital heart diseases. Surgical treatment of TOF in recent years has achieved good short-term results, while the long-term results have been compromised by the pulmonary valve regurgitation. Once the radical repair indicated, usually there are two ways to enlarge the stenotic, hypoplastic right ventricular outflow tract and/or pulmonary valve: augmentation of right ventricular outflow tract and transannular patch to enlarge right ventricular outflow tract and pulmonary trunk. A ventriculotomy should be extended across the pulmonary annulus, which results in the progressive enlargement of right ventricle secondary to the pulmonary regurgitation. Consequently, right ventricular dysfunction and arrhythmia may occur. In order to prevent postoperative pulmonary regurgitation, we have used a new technique to reconstruct right ventricular outflow tract since Oct. 2009. When we perform the transannular incision, we keep the pulmonary annular intact and create a pulmonary valve biorifice for reconstruction of right ventricular outflow tract.

## Methods

### Clinical data

There were 53 cases of TOF (the observation group: patients with pulmonary stenosis and the diameter of the original pulmonary valve annual larger than 4 mm) that underwent the operation with the biorifice technique, And at the same period, 50 cases of TOF operation using the conventional technique were selected as controls. All details of this study had been approved and supervised by ethics committee of the 2^nd^ Xiangya Hosptial, Central South University. Written informed consent was obtained from the patient’ custodians for publication of this report and any accompanying images. The clinical Data before the operation were not significantly different (Table 1). All children had different degrees of cyanosis and the skin oxygen saturations varied from 70% to 95% at rest conditions, and 26 of them had history of hypoxic episodes. Other manifestations included right ventricular hypertrophy as shown by electrocardiogram, and clear lung fields and boot shaped heart shadow as revealed by X-ray. The diagnosis of TOF for all children was confirmed by echocardiography and CT cardiac imaging examination. In addition, 3 cases underwent cardiac catheterization angiography.

**Table 1:** Preoperative dates from 2 groups

	**Observation group**	**Control group**	**P Value**
Gender (Male/Female)	28/25	23/27	>0.05
Month age (median)	31(10-45)	35 (11-44)	>0.05
Body weight (kg median)	13.1(6.5-17.6)	12.8 (6.2-17.5)	>0.05
Hemoglobin (g/L)	144±19	150±22	>0.05
Oxygen blood pressure (mmHg)	68±9	68±7	>0.05
Pulmonary artery index (mm^2^/m^2^)	122±15	129±17	>0.05
Left ventricular end diastolic volume index (ml/m^2^)	33±4	35±4	>0.05

### Surgical procedure

**Figure 1:**
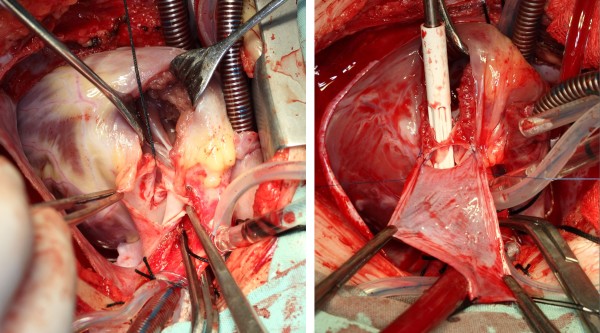
**Using autologous pericardial patch to enlarge the pulmonary artery and the right ventricular outflow tract.** Left figure shows the integrity of original pulmonary valve annular (held by a silk thread, as the first orifice). The sucker for right heart (right figure, white color) was inserted into the original pulmonary annular. And the space between the pericardium and the original pulmonary annular formed the second orifice.

All the operations were performed under general anesthesia and under support of moderate hypothermic cardiopulmonary bypass. After a median sternotomy and opening of the pericardium, the main pulmonary artery, as well as the left and right pulmonary arteries were mobilized. The aorta and both vena cava were cannulated and cardiopulmonary bypass was established and then launched. During the operation, the body temperature and infusion flow were adjusted based on the amount of blood returned from pulmonary artery. The ascending aorta was cross clamped and cold cardioplegia was administered every 30 min in the aortic root. The right atrium was opened with an antero-inferior incision, and a vent was inserted in the left atrium through the foramen ovale or a window of atrial septal. A longitudinal incision was carried on the right ventricular outflow tract. The ventricular septal defect was closed through approach of the right atrial-tricuspid and/or the right ventricular outflow tract incision. The development of the pulmonary system including pulmonary annular, main trunk and left and right branches were checked meticulously. For the cases in the observation group, because of varying degrees of stenosis of pulmonary annular, the longitudinal incision on the right ventricular outflow tract was extended, passing through pulmonary annular, towards proximal part of right and left pulmonary artery. When performing this transannular incision, one should not perform valvotomy, and careful attention must be paid to mobilize the fibrous tissue of the pulmonary valve annular and to cut the valve leaflets adhesion precisely. The integrity of the original orifice of the pulmonary valve was retained, as the first orifice (Figure 1, left). We prefered to reconstruct right ventricular outflow tract and pulmonary artery with autologous pericardial patch. When the sutures for transannular patch were finished, the space between the pericardium patch and the original pulmonary annular formed the second orifice (Figure 1, right). During the ventricular systolic ejection phase, pericardial patch portion of second orifice moves outward, resulting in the second orifice opening. And during the ventricular diastolic phase, the second orifice pericardial patch fit on the pulmonary valve ring, which causes the second orifice to close. Thus, the periodical movement of the pericardial portion of the second orifice coincided with the opening and closing of the original pulmonary valve, and this cooperation of movement contributes to minimize the pulmonary regurgitation. All patients in the control group were treated with conventional technique to enlarge the right ventricular outflow tract and pulmonary artery with single transannular patch. The summation of diameter of the two pulmonary annular orifices in the observation group was slightly greater than the diameter of single orifice in the control group. All other present malformations were repaired at the same operation time. And after operation, the postoperative treatment was not significantly different.

### Statistical analysis

Data were expressed as mean ± SE and median. Staxtistical Product and Service Solutions 14.0 software (SPSS Institute) was used for all analysis. A X^2^ test or independent-sample t-test was performed to check for differences between the two groups. The critical alpha level for these analysis was set at p<0.05.

## Results

**Figure 2:**
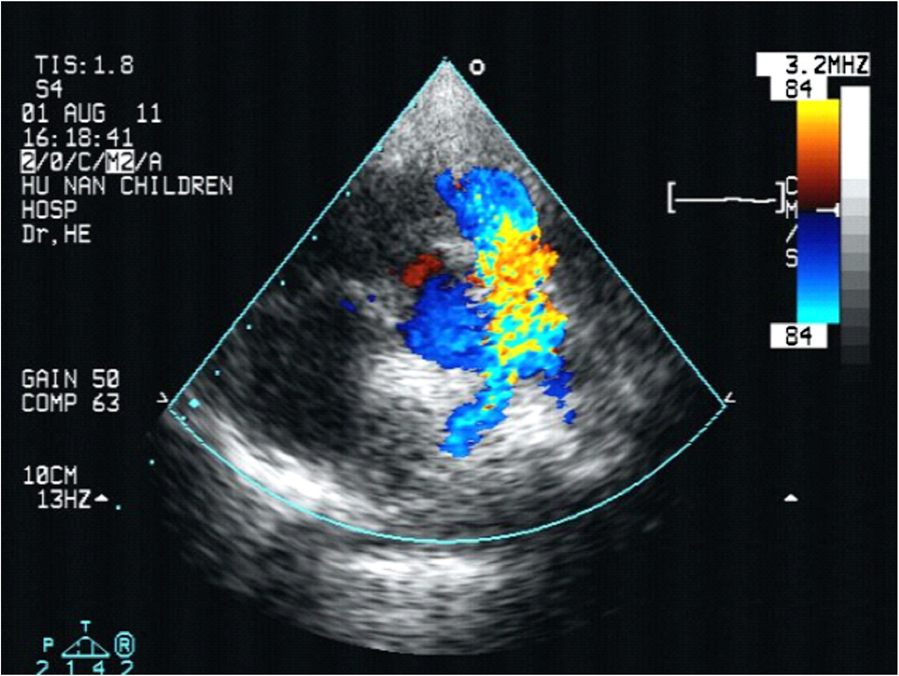
The echocardiographic findings of a case from the observation group 1 week after the operation shows the blood passing through the pulmonary valve with a slightly higher speed and no pulmonary regurgitation.

The age and the body weights were not significantly different between the two study groups (Table 1). The mean bypass and cross clamp times were similar with groups, and the postoperative ventilator support time was not significantly different between two groups. The duration of staying in intensive care unit for the observation group was shorter than the control group. The general condition of postoperative pleural effusion was improved in the observation group (Table 2). Post- operation, in the observation group, only 2 cases had large amounts of pleural effusion, 1 case had a mid-level amount and 8 cases had a little amount of pleural effusion. While in the control group, there was 1 case of a large amount of effusion, 5 cases of mid-level effusion and 17 cases of a small amount of pleural effusion. One week after the operation, all patients were re-checked by echocardiography and no pulmonary valve stenosis was observed. In the observation group, 30 cases had excellent pulmonary valve function without any regurgitation (Figure 2), 15 cases had mild regurgitation and 8 cases had moderate regurgitation. While in the control group, only 14 cases had excellent pulmonary valve function without regurgitation, and 16 cases had mild regurgitation, 17 cases had moderate regurgitation. 33 cases from the observation group were followed-up 6 months later and mild pulmonary regurgitation was found in 3 cases, and 18 cases of the observation group (including the 3 cases who had mild pulmonary regurgitation at 6 months follow-up) were rechecked 1 year later and no pulmonary regurgitation was found. While 45 cases from the control group were followed-up 6 months later, 1 case had severe pulmonary regurgitation, 22 cases had moderate-mild regurgitation; and 1 year later, 26 cases of the control group rechecked, 3 cases had severe regurgitation and 11 cases had moderate-mild regurgitation.

**Table 2:** Two groups of children’s postoperative data

	**Observation group**	**Control group**	**P values**
Cardiopulmonary bypass time (min)	68±19	64±27	>0.05
Aortic cross clamp time (min)	39±10	40±15	>0.05
Mechanical ventilation time (h)	48±23	62±35	>0.05
ICU residence time (d)	5.5±2.2	8.5±5.0	<0.05
Pleural effusion (No / small / medium / large)**	42/8/1/2	27/17/5/1	<0.05

**Total pleural effusion of postoperation. small: ≦10 mL/kg, medium: 10~30 mL/kg, large: ≧30 mL/kg. (Exclude bleeding of operation).

## Discussion

Tetralogy of Fallot, accounts for 12%-14% of all congenital heart diseases, is one of the most common complex cyanotic congenital heart diseases. The pathological anatomic characteristics for a tetralogy include ventricular septal defect, right ventricular outflow tract obstruction, overriding of aorta, and right ventricular hypertrophy. Recently, the one stage repair for a tetralogy was performed in many heart centers around the world and led to a satisfactory short-term outcome in children, as reported by some centers [1]. Because of the presence of the pulmonary valve dysplasia, a transannular patch to enlarge the narrowed right ventricular outflow tract and pulmonary artery is required. The main concern for this transannular patch is that the integrity of pulmonary valve will be damaged, which results in postoperative pulmonary regurgitation. Thus, the main shortfall of the conventional transannular technique is the poor long-term outcome, and therefore, the chances of reoperation is greater [2]. Long-term pulmonary valve regurgitation can lead to further right ventricular hypertrophy and right heart failure [3]. And [1] in such conditions, additional surgical interventions (pulmonary valve replacement surgery) may be required to correct pulmonary regurgitation for the purpose of the long term outcome [4]. It is estimated that about 10% to 15% or even higher percentages of patients may have severe pulmonary regurgitation 20 years after the first operation, and pulmonary valve replacement may be required for those patients [5,6].

How to effectively reduce pulmonary regurgitation and reduce the necessity for a pulmonary valve replacement is one of the study focuses of cardiac surgeons. Some cardiac surgeons add single or double artificial leaflets on the autologous patch used to reconstruct the right ventricular outflow tract [4]. The added leaflets were made by autologous pericardium, or polytetrafluoroethylene (PTFE), or other kinds of biological materials. However, all of these materials have disadvantages. For instance, PTFE is not very compatible with heart tissue and is very difficult to suture on to the surface of pericardium, which limits its further use. Other kinds of biological materials including bovine jugular vein (with valve), allograft aortic valve and pulmonary valve are used commonly. The initial results of the clinical application showed that these materials had relatively better tissue compatibility and less pulmonary regurgitation compared to PTFE. As the bovine jugular vein is easy to obtain, its usage is more common than the use of allograft of aortic or pulmonary valve. Although those biological materials have some advantages when compared with PTFE, degeneration and calcification are the main disadvantages for biological material [7]. Meanwhile, studies related to anti-degeneration and/or anti-calcification is another important direction of current research [8].

**Figure 3:**
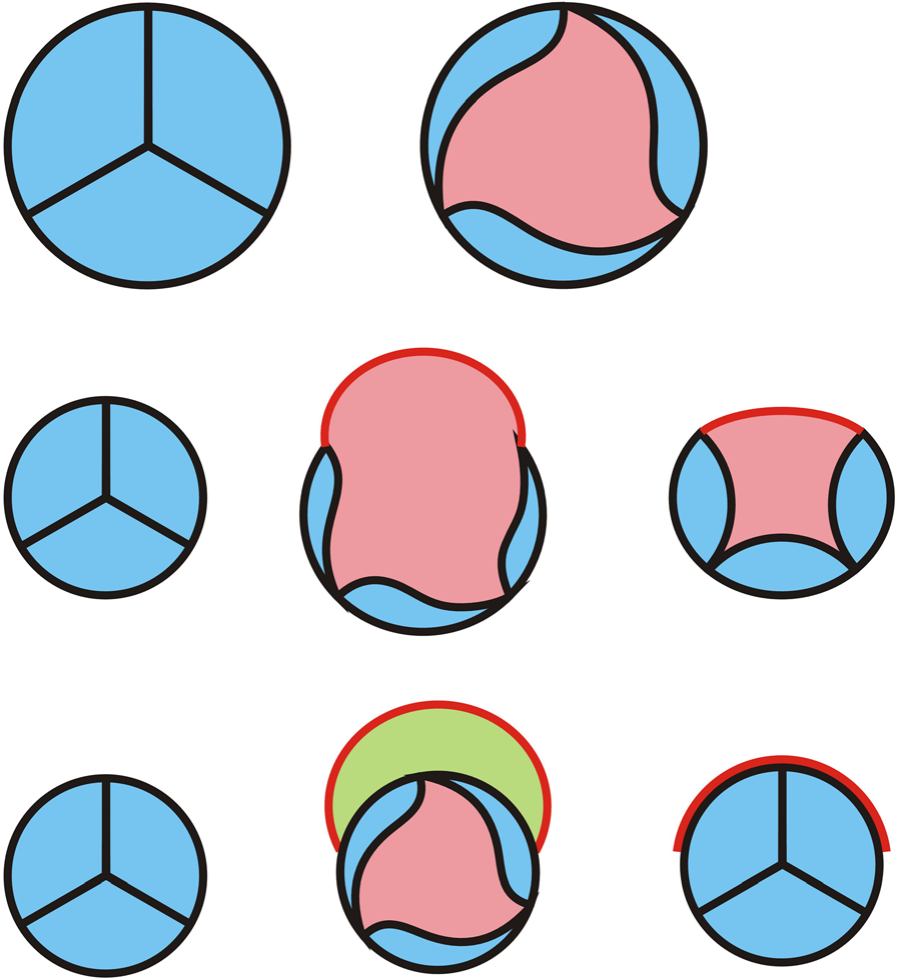
The first line of this schematic shows the movement of normal pulmonary valve, The second line shows the movement of stenotic pulmonary valve enlarged with conventional transannular patch, The third line shows the original pulmonary valve been reserved and the second pulmonary orifice be constructed by the original annulus and pericardium.

In the last 20 years, the general view of the surgical correction of tetralogy has changed. Many surgeons used to emphasize the complete release of outflow tract obstruction, while more and more cardiac surgeons try to retain the pulmonary valve even in the case of residual moderate outflow tract obstruction [9]. By retaining the pulmonary valve, the longer-term outcome for patients have improved, as the pulmonary valve regurgitation effects are reduced. The outflow tract integrity helps to avoid late outflow tract patch aneurysm. Based on this view, we retained the original pulmonary valve and reconstructed the right ventricular outflow tract and pulmonary artery with "biorifice" angioplasty. One key point of the biorifice technique is to reserve the function of original pulmonary valve. It is very important to cut the adhesive pulmonary leaflets precisely in case there are dysplasia leaflet adhesions present. Besides the original pulmonary orifice, the forward blood stream can pass through the “second channel” which is constructed from the original pulmonary annular and the autologous pericardium. The opening and closing of the second channel, or the second orifice, is driven by the pressure gradient between the right ventricle and the pulmonary artery. During diastole, the right ventricular negative pressure effect (Bernouli effect) causes the pericardial patch to move towards the original pulmonary annular and results in the closure of the "second channel". Both the reserved original pulmonary valve function and the movement of the second channel are the anatomic mechanism of anti-regurgitation (Figure 3).

In this study, we selected patients with pulmonary stenosis which must be enlarged and while the diameter of the original pulmonary valve annual was not too small. In our institution, there were more than 120 patients with TOF admitted at this study period and all those patients had been screened for the indication of biorifice technique. And approximately 40% of them were suitable for this technique. If the diameter less than 4 mm means the bi-orifice technique may not result beneficial for these patients. Due to some economic and political reasons, patients with congenital heart disease in eastern world may not come to hospital early, and this is the reason why the mean ages of this study are older when compared with studies from western world.

All cases in this study were followed-up for up to one year and no early deaths were reported. The initial findings of our study were promising, however, due to the low numbers of patients involved and the absence of longterm follow ups, further studies are necessary to to fully evaluate the benefits of the biorifice technique.

## Conclusions

The biorifice technique of pulmonary valve can reduce pulmonary valve regurgitation, reduce postoperative pleural effusion, and improve the early surgical outcome.

## Competing interests

The authors declare that they have no competing interests.

## Authors’ contributions

JY and WZ carried out the design of the operative procedure for bi-orifice technique, perfomed all the operations and wrote the manuscript. LX carried out the Data collection. LX carried out the figures collection. XW and YY carried out the Data analysis. All authors read and approved the final manuscript.
